# Final overall survival in JO22903, a phase II, open-label study of first-line erlotinib for Japanese patients with *EGFR* mutation-positive non-small-cell lung cancer

**DOI:** 10.1007/s10147-016-1039-0

**Published:** 2016-09-22

**Authors:** Noboru Yamamoto, Koichi Goto, Makoto Nishio, Kenichi Chikamori, Toyoaki Hida, Makoto Maemondo, Nobuyuki Katakami, Toshiyuki Kozuki, Hiroshige Yoshioka, Takashi Seto, Kosei Tajima, Tomohide Tamura

**Affiliations:** 1 Department of Thoracic Oncology, National Cancer Centre Hospital, Chuo-ku, Tokyo Japan; 2 Department of Thoracic Oncology, National Cancer Centre Hospital East, Kashiwa, Chiba Japan; 3 Thoracic Oncology Centre, The Cancer Institute Hospital of the Japanese Foundation for Cancer Research, Koto-ku, Tokyo Japan; 4 Oncology Medicine, National Hospital Organization, Yamaguchi-Ube Medical Centre, Ube, Yamaguchi Japan; 5 Department of Thoracic Oncology, Aichi Cancer Centre Hospital, Nagoya, Aichi Japan; 6 Department of Respiratory Medicine, Miyagi Cancer Centre, Natori, Miyagi Japan; 7 Integrated Oncology, Institute of Biomedical Research and Innovation Hospital, Kobe, Hyogo Japan; 8 Department of Thoracic Oncology, National Hospital Organization, Shikoku Cancer Centre, Matsuyama, Ehime Japan; 9 Department of Respiratory Medicine, Kurashiki Central Hospital, Kurashiki, Okayama Japan; 10 Department of Thoracic Oncology, National Kyushu Cancer Centre, Fukuoka, Fukuoka Japan; 11 Clinical Research Planning Department, Chugai Pharmaceutical Co. Ltd, Chuo-ku, Tokyo Japan; 12 Thoracic Center, St Luke’s International Hospital, Chuo-ku, Tokyo Japan

**Keywords:** Erlotinib, *EGFR* mutations, Non-small-cell lung cancer (NSCLC), First line, Japanese patients, Overall survival

## Abstract

**Background:**

In Japan, the clinical efficacy of erlotinib monotherapy in *epidermal growth factor receptor (EGFR)* mutation-positive non-small-cell lung cancer was demonstrated in the phase II JO22903 trial, which reported a median progression-free survival of 11.8 months. Here we report final overall survival data from JO22903.

**Methods:**

JO22903 (JapicCTI-101085) was a single-arm, multicenter, phase II, open-label, non-randomized study of first-line erlotinib monotherapy in *EGFR* mutation-positive non-small-cell lung cancer. Eligible patients (≥20 years) with stage IIIB/IV or recurrent non-small-cell lung cancer and confirmed activating mutations of *EGFR* (exon 19 deletion or L858R point mutation in exon 21) received oral erlotinib 150 mg/day until disease progression or unacceptable toxicity. The primary endpoints were progression-free survival and safety; overall survival was a secondary endpoint.

**Results:**

At the final analysis, 102 patients were included in the modified intent-to-treat population and 103 in the safety population. Median follow-up was 32.3 months. Median overall survival was 36.3 months (95 % confidence interval 29.4–not reached). Subgroup analyses of overall survival suggested that the presence of brain metastases was a negative prognostic factor (median overall survival 22.7 months, 95 % confidence interval 19.6–29.4). The impact on overall survival of using versus not using EGFR tyrosine kinase inhibitors in any line of treatment following disease progression was unclear (median 32.8 versus 36.3 months, respectively). No new safety issues were observed.

**Conclusion:**

In this survival update, single-agent erlotinib achieved a median overall survival of more than 3 years in patients with *EGFR* mutation-positive non-small-cell lung cancer.

## Introduction

In non-small-cell lung cancer (NSCLC), platinum doublet chemotherapy followed by second-line docetaxel monotherapy [1] or pemetrexed maintenance therapy following first-line platinum doublet chemotherapy [2] prolongs survival outcomes for patients with non-squamous NSCLC. Based on the efficacy of these treatments, it has been anticipated that they will improve long-term survival of patients with epidermal growth factor receptor (*EGFR*) mutation-positive NSCLC after the administration of EGFR tyrosine kinase inhibitors (TKIs).

The treatment of NSCLC has changed considerably in recent years. Following the discovery of the pivotal oncogenic role of *EGFR* in unselected NSCLC [3, 4], the subsequent development of EGFR TKIs provided new therapeutic options for the treatment of this disease. Greater understanding of tumor biology has since led to the discovery that tumors with sensitizing *EGFR* mutations, particularly the somatic mutations in *EGFR* exons 19 and 21, respond favorably to EGFR TKIs compared with chemotherapy [5]. To reflect this, EGFR TKIs are recommended in clinical treatment guidelines for NSCLC.

Currently, gefitinib, erlotinib and afatinib are the only EGFR TKIs approved (US Food and Drug Administration, EU and Japan) for the treatment of *EGFR* mutation-positive NSCLC [6, 7]. These approvals were supported by data from several phase III clinical trials, which consistently reported that EGFR TKIs demonstrate significant progression-free survival (PFS) benefits compared with standard chemotherapy [8]. Median PFS with first-line gefitinib in *EGFR* mutation-positive NSCLC ranged between 9.6 and 10.4 months in the pan-Asian IPASS study of gefitinib versus carboplatin/paclitaxel [9], the Japanese NEJ002 study of gefitinib versus carboplatin-paclitaxel [10], and the WJTOG3405 study of gefitinib versus cisplatin/docetaxel [11]. However, despite similar PFS results with gefitinib in these studies, median OS was not consistent; the IPASS study reported a median OS of 21.6 months with gefitinib [9], whereas a longer median OS of 27.7 months was published in the NEJ002 study [10] and a median OS of 34.8 months was reported with gefitinib in the Japanese WJTOG3405 study [11].

Median OS with erlotinib in *EGFR* mutation-positive NSCLC was 22.7 months in the phase III OPTIMAL study of erlotinib versus gemcitabine plus carboplatin [12], and 22.9 months in the phase III EURTAC study of erlotinib versus chemotherapy [13]. However, as these two studies were conducted outside of Japan, the median OS with erlotinib in Japanese patients with *EGFR* mutation-positive NSCLC is currently unknown. PFS for the single-agent erlotinib arm of the Japanese phase II JO25567 study was 9.7 months [14], which was similar to the 11.8 months median PFS (primary endpoint) reported for the phase II Japanese JO22903 study [15]. Here, we report final OS data with erlotinib monotherapy in the JO22903 study and present exploratory analyses of OS with respect to *EGFR* mutation subtype. We also evaluated whether OS was impacted by the use of post-progression therapy.

## Patients and methods

### Study design and patients

JO22903 (JapicCTI-101085) was a phase II, single-arm, multicenter, open-label, non-randomized study of first-line erlotinib monotherapy for the treatment of *EGFR* mutation-positive NSCLC. Full study design information has been previously published [15]. Briefly, the study was conducted at 25 centers in Japan. Patients were aged ≥20 years with stage IIIB/IV or recurrent NSCLC, with no prior chemotherapy, Eastern Cooperative Oncology Group performance status of 0 or 1, and tumors harboring confirmed activating mutations of *EGFR* (exon 19 deletions or L858R point mutations in exon 21). Patients were excluded if they had symptomatic brain metastases or if they had co-existence or history of interstitial lung disease (ILD). After discontinuation of the protocol treatment, patients were treated at the investigators discretion.

JO22903 was carried out in accordance with the Declaration of Helsinki and also the Japanese Good Clinical Practice Guidelines. All patients provided written informed consent for study participation. The study protocol was approved by the local ethics committees.

### Procedures

Full treatment procedures have been published previously [15]. Briefly, patients received oral erlotinib 150 mg/day until disease progression (PD) or unacceptable toxicity. Treatment was interrupted if ILD was suspected; for patients with confirmed ILD diagnosis, erlotinib was discontinued immediately. In cases of gastrointestinal perforation or any grade 4 adverse events (AEs), erlotinib was discontinued. Patients were screened for *EGFR* mutations in a local or central laboratory; *EGFR* mutation status was determined using Scorpion ARMS as described previously [15]. Lung and abdominal scans [computed tomography (CT)/magnetic resonance imaging (MRI)] were mandatory at baseline and during treatment until PD. Brain scans were mandatory at baseline (CT/MRI).

### Assessments

Tumor response was assessed by an independent review committee (IRC) using Response Evaluable Criteria in Solid Tumours (RECIST) version 1.0. The analysis of safety parameters was descriptive; safety was assessed according to the Medical Dictionary for Regulatory Activities (version 14.0) preferred terms and tabulated by grade. All patients who received at least one dose of study treatment were included in the safety population. A modified intent-to-treat (ITT) population was used for the efficacy analysis, which included all patients from the safety population without major protocol violations.

### Study endpoints

The co-primary endpoints were PFS in the modified ITT population as assessed by IRC according to RECIST version 1.0, and safety. Secondary endpoints included OS and overall response rate.

### Statistical analyses

Kaplan–Meier methodology was used to estimate median and 95 % confidence intervals (CI) for OS, and hazard ratios (HR) were estimated by the use of a Cox model. CI limits were calculated according to the Greenwood method.

## Results

### Patients

Patients were enrolled between April 2010 and October 2010. Median follow-up was 32.2 months. At the time of this final analysis, 103 patients with confirmed *EGFR* mutations were included in the study. The safety population comprised all 103 patients whilst the modified ITT population comprised 102 patients; one patient was excluded due to a major protocol violation (receipt of incorrect study medication) after enrolment.

Baseline patient characteristics have been previously published [15]. Briefly, the majority of patients were female (*n* = 70), with stage IV disease (*n* = 74), adenocarcinoma histology (*n* = 102), and were never-smokers (*n* = 59).

### Efficacy analyses

**Fig. 1:**
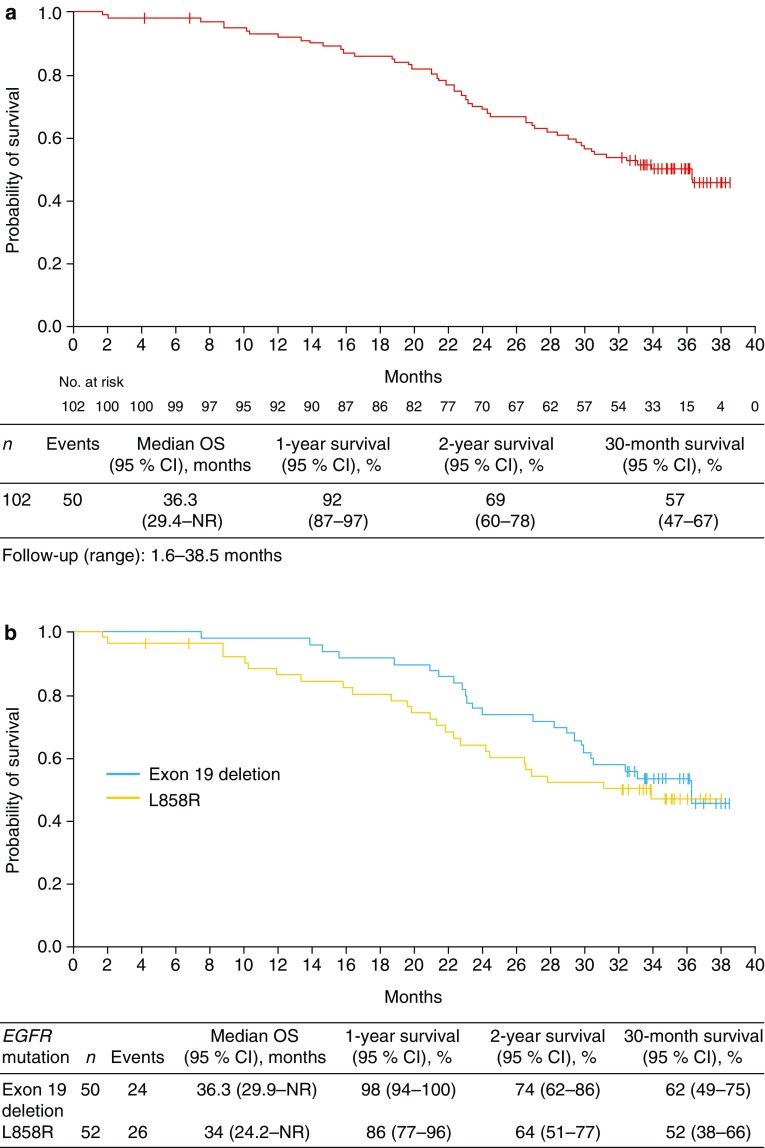
Overall survival with **a** erlotinib monotherapy in the modified ITT population and **b** by *EGFR* mutation type

In the modified ITT population at the updated data cut-off, median OS with first-line erlotinib was 36.3 months (95 % CI: 29.4–not reached [NR]) based on the occurrence of 50 events. The 1-year survival rate was 92 % (95 % CI 87–97), the 2-year survival rate was 69 % (95 % CI 60–78) and the 30-month survival rate was 57 % (95 % CI 47–67) (Fig. 1a). Univariate subgroup analyses showed shorter OS in patients with brain metastases at baseline (median OS 22.7 months, 95 % CI 19.6–29.4) and in those with a T790M *EGFR* mutation (median OS 20.0 months, 95 % CI 15.8–24.2) (Table 1). When analyzed by the specific type of *EGFR* mutation, median OS was 36.3 months (95 % CI 29.9–NR) versus 34.0 months (95 % CI 24.2–NR), respectively, for patients with exon 19 deletion versus exon 21 L858R point mutation (HR 0.77, 95 % CI 0.44–1.35, *p* = 0.3662) (Fig. 1b).

**Table 1:** Subgroup analysis of median overall survival

Characteristics	*n*	Events	Median OS (months)	95 % CI
Gender				
Female	69	33	36.3	29.4–NR
Male	33	17	34.0	23.4–NR
Age				
<75 years	88	43	36.3	28.3–NR
≥75 years	14	7	31.2	18.6–NR
Stage				
IIIB/IV	77	43	31.2	26.5–NR
Recurrence	25	7	NR	28.3–NR
Smoking status				
Yes	44	24	31.2	23.4–NR
No	58	26	36.3	29.8–NR
*EGFR* mutation status				
Exon 19 deletion	50	24	36.3	29.8–NR
L858R	50	24	34.0	22.7–NR
L858R + T790M	2	2	20.0	15.8–24.2
Brain metastases				
Yes	21	16	22.7	19.6–29.4
No	81	34	NR	32.4–NR

CI confidence interval, *EGFR* epidermal growth factor receptor, *OS* overall survival, *NR* not reached

**Fig. 2:**
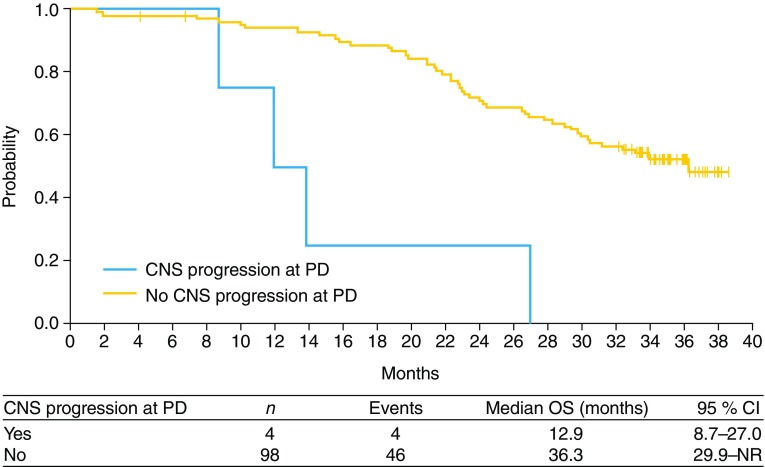
Overall survival by CNS progression

Four patients had PD with central nervous system (CNS) progression (Table 2; Fig. 2). Median OS was shorter in patients with CNS PD compared with those without (12.9 months [95 % CI 8.7–27.0] versus 36.3 months [95 % CI 22.9–NR]).

**Table 2:** Characteristics of patients who had CNS progression in erlotinib treatment

Number	Age (years)	Gender	ECOG PS	*EGFR* mutation	Baseline CNS metastases	Erlotinib dose at PD (mg)	PFS (days)	OS (days)
1	50	M	1	19 del	No	100	106	420
2	55	M	1	19 del	Yes	150	335	823
3	55	F	0	L858R	No	150	168	363
4	73	F	1	L858R	Yes	150	80	266

*CNS* central nervous system, *del* deletion, *ECOG PS* Eastern Cooperative Oncology Group performance status, *EGFR* epidermal growth factor receptor, *F* female, *M* male, *OS* overall survival, *PD* progressive disease, *PFS* progression-free survival

### Post-progression therapy

**Fig. 3:**
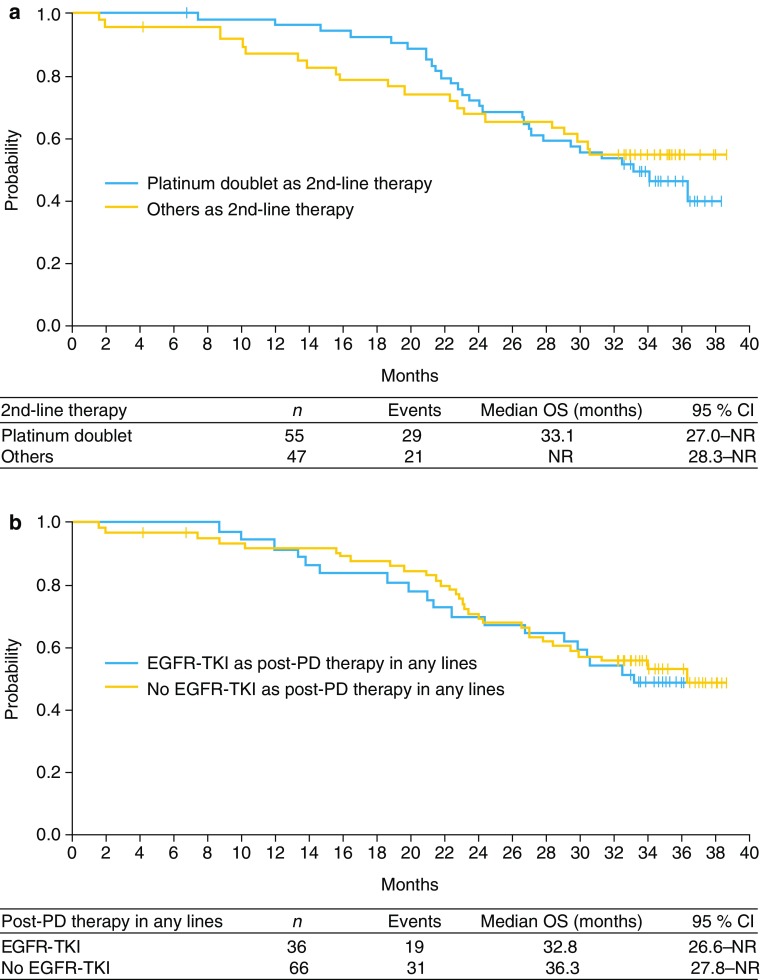
Overall survival by post-PD therapy with **a** second-line platinum doublet chemotherapy and **b** EGFR TKI in any line

Following PD, the majority of patients went on to receive either platinum doublet chemotherapy, with or without bevacizumab (*n* = 60), further EGFR TKIs (*n* = 35), or single-agent chemotherapy (*n* = 39) (Table 3). In terms of second-line therapy, median OS was similar in patients who were treated with platinum doublet chemotherapy or other types of therapy [median OS 33.1 months (95 % CI 27.0–NR) versus NR, respectively; Fig. 3a]. The use of further EGFR TKIs in any line of treatment following PD also had no apparent impact on OS compared with not using an EGFR TKI as post-PD therapy in any line [median OS 32.8 months (95 % CI 26.6–NR) versus 36.3 months (95 % CI 27.8–NR), respectively; Fig. 3b].

**Table 3:** Therapies given upon disease progression (eight patients were receiving study treatment at data collection. Information was unavailable for ten patients)

Therapy (*n*)	Second-line therapy	All lines of treatment
Platinum doublet	46	60
Without bevacizumab	31	41
With bevacizumab	15	21
EGFR TKI	30	35
Erlotinib	21	25
Gefitinib	8	14
Erlotinib + tivantinib	1	1
Erlotinib + pemetrexed	0	1
Gefitinib + pemetrexed	0	1
Single-agent chemotherapy	7	39
Docetaxel + bevacizumab	3	4
Pemetrexed	3	15
Docetaxel	1	24
Pemetrexed + bevacizumab	0	1
Platinum doublet + EGFR TKI	1	2
With erlotinib	1	2
Others	0	16

*EGFR TKI* epidermal growth factor receptor tyrosine kinase inhibitor

### Safety

The safety profile of erlotinib did not change at this data update (Table 4) and was as previously reported [15]. The most common all grade treatment-related AEs were rash (82.5 %) and diarrhea (79.6 %), and the most common grade ≥3 treatment-related AEs were rash (14.6 %) and an increase in alanine aminotransferase (8.7 %).

**Table 4:** Treatment-related adverse events, all grades (≥30 %) and grade ≥3 (≥5 %)

	All grades (≥30 %)	Grade ≥3 (≥5 %)
	*n* (%)	*n* (%)
Rash	85 (82.5)	15 (14.6)
Diarrhea	82 (79.6)	0 (0.0)
Dry skin	82 (79.6)	0 (0.0)
Paronychia	69 (67.0)	0 (0.0)
Stomatitis	65 (63.1)	0 (0.0)
Pruritus	67 (65.0)	0 (0.0)
Decreased appetite	35 (34.0)	0 (0.0)
ALT increased	33 (32.0)	15 (14.6)

*ALT* alanine aminotransaminase

## Discussion

EGFR TKIs are the standard of care for the first-line treatment of *EGFR* mutation-positive NSCLC [6, 7]. In Japan, the phase II, single-arm JO22903 study demonstrated efficacy of erlotinib monotherapy in *EGFR* mutation-positive NSCLC, with a reported median PFS of 11.8 months [15]. In this updated analysis of the JO22903 study, the 30-month OS rate was 57 % (95 % CI 47–67) and median OS was 36.3 months (95 % CI 29.4–NR). These findings represent a more favorable OS than observed in previous studies of first-line erlotinib in *EGFR* mutation-positive NSCLC outside Japan (median OS range 22.9–26.3 months [12, 13, 16]), and are in line with results from prospective studies of other EGFR TKIs in Japanese populations (median OS range 27.7–34.8 months [10, 11]). Recently, a median OS of 46.9 months was reported for Japanese patients who received afatinib in the LUX-Lung 3 study [17], which was longer than that observed in the entire study population [18]. Across these studies, the median PFS values observed in Japanese and global populations were very similar, at approximately 1 year [10–13, 16–18]. Thus, it seems that the current treatment landscape in Japan may be contributing to a longer OS compared with non-Japanese populations, and that OS in Japanese populations can reasonably be expected to reach beyond 3 years.

Although patients with brain metastases have a poor prognosis, which is reflected by the shorter median OS for this subgroup, the findings of this present analysis suggest that erlotinib could be considered effective for patients with brain metastases, as only four patients had CNS progression. This finding is consistent with the phase II ASPIRATION study in Asian patients, which reported that just 4.3 % of patients treated with post-PD erlotinib had new brain lesions [19]. This role for EGFR TKIs has also been observed in populations not restricted to Japanese or Asian patients [20–22]. A case series of 15 patients with NSCLC with *EGFR* mutations and CNS metastases who received cerebrospinal fluid concentration (CSF) examinations during EGFR TKI treatment provides further evidence to support this conclusion. In this case series, CNS response rate was 57 % with a favorable penetration rate of erlotinib in the CSF [23]. The penetration rate of erlotinib may be dependent on its affinity for p-glycoprotein, which pumps drugs out of the CNS. These findings suggest that erlotinib has a favorable pharmacokinetic profile as a treatment option for patients with brain metastases.

Patients with an exon 19 deletion appeared to have longer OS in our analysis than those with exon 21 L858R *EGFR* mutation-positive NSCLC. This is similar to the results of a meta-analysis of seven trials (*n* = 1649), which concluded that patients with an exon 19 deletion had better efficacy outcomes than patients with exon 21 L858R *EGFR* mutation-positive NSCLC, regardless of which EGFR TKI they received [24]. These data suggest that patients with exon 19 deletion and exon 21 *L858R* EGFR mutation are clinically distinct populations that should be evaluated further.

In the present study, there was no apparent difference in OS according to subsequent treatments. Median OS was similar for patients who received EGFR TKIs as post-PD therapy (*n* = 36), which were mainly continuous erlotinib administration following RECIST PD (*n* = 21) (Table 3), and for those who did not. In contrast to our findings, in a retrospective study of patients with activating *EGFR* mutations (*n* = 123) who were treated with EGFR TKIs, OS showed a trend in favor of continuing versus discontinuing EGFR TKI treatment following RECIST PD (33.0 versus 21.2 months, respectively; *p* = 0.054) [25]. Furthermore, a retrospective clinical modeling study that evaluated the usefulness of EGFR TKI failure pattern for selecting subsequent management, suggested that the efficacy of EGFR TKI continuation differed between patients with gradual progression, local progression, and dramatic progression [26]. Thus, one hypothesis for the inconsistency between studies is the difference in the EGFR TKI failure pattern. Meanwhile, in the present study, various EGFR TKIs were used as post-PD therapy (i.e., erlotinib beyond progression, erlotinib re-challenge after another treatment, and other therapies), which should be noted as one of the limitations. As effective post-PD therapy options are important for patients with disease recurrence, any benefit of EGFR TKI re-administration or continuation after PD requires further study.

At this updated analysis, no new safety signals for erlotinib were observed; single-agent erlotinib was well tolerated and had an acceptable and manageable safety profile in *EGFR* mutation-positive NSCLC. The safety profile of erlotinib was also in line with previous studies of first-line erlotinib [13], with the most common AEs being rash and diarrhea.

In conclusion, single-agent erlotinib resulted in a median OS of 36.3 months in the first-line treatment of *EGFR* mutation-positive NSCLC. Subgroup analyses of OS suggested that the presence of brain metastases was a negative prognostic factor, as these patients had shorter median OS compared with other subgroups. No further differences in OS between specific *EGFR* subgroups were observed. Although many patients went on to receive additional EGFR TKI therapy following progression, there was no significant difference in median OS for patients who received EGFR TKI as post-PD therapy compared with those who did not. The findings of this single-arm study should be validated in randomized controlled trials.
